# A JNK‐Regulated and IL‐1β‐Dependent Astrocyte–Neuron Signaling Pathway in the Spinal Dorsal Horn is Essential for Stress‐Induced Hyperalgesia

**DOI:** 10.1155/np/2791699

**Published:** 2026-03-15

**Authors:** Jian Qi, Chen Chen, Qian Gao

**Affiliations:** ^1^ Department of Orthopedics, The 960th Hospital of PLA, Jinan, 250031, China; ^2^ Department of Pharmacy, The Second Hospital of Shandong University, Jinan, 250031, China, sdey.net; ^3^ Jinzhou Medical University, The 960th Hospital of The Chinese People’s Liberation Army Joint Logistic Support Force Postgraduate Training Base, Jinan, 250031, China, jzmu.edu.cn

**Keywords:** astrocyte, complete Freund’s adjuvant, IL-1β, JNK, neurons, NR2B, post-traumatic stress disorder, rat, single-prolonged stress, spinal dorsal horn

## Abstract

Various forms of mild stress may exacerbate pain in patients with chronic pain disorders, though the underlying mechanism remains unclear. Astrocyte activation in the spinal dorsal horn plays a predominant role in stress and pain. The present study investigated the neuron–astrocyte interactions in the spinal dorsal horn in post‐traumatic stress disorder (PTSD)‐induced hyperalgesia using a single‐prolonged stress (SPS) model, a Complete Freund’s Adjuvant (CFA) model and an SPS + CFA model. Animals were tested for mechanical withdrawal threshold (MWT) of the paw after SPS, CFA and SPS + CFA. SPS + CFA group induced significantly increased mechanical allodynia compared with the SPS or CFA group. We tested the hypothesis that IL‐1β contributes to signaling between astrocytes and neurons in stress‐induced hyperalgesia (SIH). Immunohistochemical data showed that there was an upregulation of glial fibrillary acidic proteins (GFAPs, a marker of astrocyte) and Fos (a marker of neuron) in SIH. Immunohistochemical data showed specific localization of IL‐1β to astrocyte, but not to microglia and neurons and a neuronal localization of the IL‐1β receptor (IL‐1RI) with NMDAR2B (NR2B). Enzyme immunoassay analysis showed that IL‐1β release was dependent on c‐Jun N‐terminal kinase (JNK) activation in astrocyte. The JNK inhibitor SP600125 suppressed IL‐1β release. SP600125 and IL‐1RI blockade with IL‐1ra resulted in a restoration of behavioral nociceptive thresholds. Our results showed that the IL‐1β‐dependent, JNK‐regulated astrocyte–neuron signaling pathway mediated the astroglia component of pain maintenance in SIH.

## 1. Introduction

Stress is known to increase pain, a phenomenon termed “stress‐induced hyperalgesia (SIH)” [1–4]. Animal studies demonstrate that repeated swim stress and chronic restraint can induce hyperalgesia. Additionally, clinical evidence indicates that stress increases vulnerability to pain and worsens preexisting pain conditions [5–9]. Thus, it is important to understand how stress affects the development and severity of chronic pain. Post‐traumatic stress disorder (PTSD), an anxiety disorder triggered by exposure to life‐threatening traumatic events, has been associated with heightened pain sensitivity according to previous research [2]. However, the underlying mechanism that underpins this sensitivity is not fully understood.

Glia in the CNS, including the spinal cord, refers to several distinct nonneuronal cell types, including oligodendrocytes, microglia, and astrocytes [10]. Evidence suggests that glia is intimately involved in the active control of neuronal activity [11]. Glia‐neuro interaction has become a dynamic and prolific field of investigation, challenging old established concepts in the neurobiology of chronic pain [12]. Through still unknown mechanisms, glia can be activated after pain and release chemical mediators that modulate neuronal activity and synaptic strength [13–15]. Pro‐inflammatory mediators released from glia, including cytokines, chemokines and neuromodulators can act on neuronal functions but also on glial cells themselves [16]. Such reciprocal interactions between glia and neurons are thought to contribute to the activation of a neuro‐glial amplification loop leading to the amplification of the pain [17, 18]. Our previous results showed that the presence of a “crosstalk” between activated microglia and neurons in the spinal dorsal horn, which might contribute to the stress‐induced hyperactivated state, leads to an increased pain sensitivity [2].

A wide range of conditions (stimuli related to trauma, ischemia, invading pathogens, or stress) can activate astrocytes, as classically characterized by hypertrophied morphology, increased proliferation and upregulation of specific markers (glial fibrillary acidic protein [GFAP]) [19]. Astrocytes play critical roles in the pathophysiology of PTSD and other anxiety illnesses [20]. Stress could active neuroendocrine and autonomic nervous systems, which are known to be involved in the modulation of the pain pathway [21]. Stress hormones may either inhibit the production of pro‐inflammatory cytokines or boost immune responses via peripheral production of pro‐inflammatory cytokines [22]. Pro‐inflammatory cytokines in the CNS, which may be linked to astrocyte activation, could lead to a sustained hyperactivated state in astrocytes following stress exposure [23]. Activation of the mitogen‐activated protein kinase (MAPK) signaling pathway, which plays a pivotal role in chronic pain development and central sensitization, is intimately connected to inflammatory cascade induction [24]. The astrocyte c‐Jun N‐terminal kinase (JNK) MAPK subfamily critically contributes to neuropathic pain development by controlling the expression of pro‐inflammatory mediators (TNF‐α, IL‐1β, and IL‐6) [25]. Notably, IL‐1β signaling appears to potentiate NMDA receptor (NMDAR) activation in neurons, representing a crucial neuroimmune interaction in pain processing [26]. These mechanisms might represent potential common therapeutic targets in SIH. Hence, we hypothesize that neuron–astrocyte interactions are involved in PTSD‐induced hyperalgesia. However, the molecular mechanisms underpinning this interaction have yet to be elucidated, along with the specific downstream cytokines involved in astrocyte–neuron crosstalk.

In the present study, we used single‐prolonged stress (SPS) to establish PTSD model. Complete Freund’s adjuvant (CFA) injection was used to promote chronic inflammatory pain. We have examined the cellular mechanisms of PTSD‐induced hyperalgesia (SPS + CFA model) with an emphasis on astrocytes‐cytokine‐neuronal interactions in the spinal dorsal horn. We hypothesize that stress activates JNK signaling in astrocytes, driving IL‐1β release. This cytokine then engages neuronal IL‐1RI to enhance NMDAR‐dependent synaptic plasticity, thereby promoting inflammatory pain pathogenesis. Our findings provide new and important evidence of stress astrocyte activation and its role in signal coupling between IL‐1β and IL‐1β receptor (IL‐1RI), leading to activity‐dependent neuronal plasticity and chronic or persistent pain.

## 2. Materials and Methods

### 2.1. Animals

Adult male Sprague–Dawley rats (200–250 g) were used in this study. In compliance with NIH guidelines, every effort was made to minimize both the number of animals used and their suffering. The study was conducted in accordance with Guidelines for the Care and Use of Mammals in Neuroscience and Behavioral Research and regulations and was approved by the Animal Ethics Committee of the Military 960th Hospital (Ethical Approval Number 200057). For surgical procedures, rats were anesthetized via intraperitoneal injection of sodium pentobarbital (45 mg/kg) dissolved in 0.9% saline.

### 2.2. Models and Surgical Groups

SPS has been suggested as an animal model for PTSD. Rats were exposed to complete restraint in disposable plastic holders for 2 h, followed by forced swimming for 20 min. After 15 min recovery, rats were exposed to diethyl ether until loss of consciousness [27]. Inflammatory pain rat model was induced by injection of 50% CFA (50 μL, Sigma, St Louis, MO, USA; 1 mg *M. tuberculosis* in 0.85 mL mineral oil and 0.15 mL mannide mono‐oleate) into the plantar surface of the right hind paw [28].

**Figure 1:**
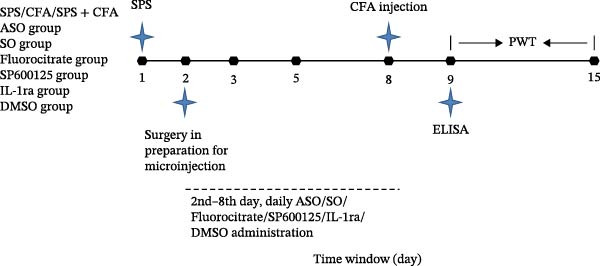
Time window for schematic diagram of experimental design. The detailed group has been shown in the diagram, and behavioral tests were conducted for each group. SPS, single‐prolonged stress; CFA, complete Freund’s adjuvant; PWT, paw withdrawal threshold; ELISA, enzyme‐linked immunosorbent assay.

As in our previous study, rats were exposed to SPS on Day 1 and/or CFA on Day 8 to establish the SPS, CFA, and SPS + CFA groups [2, 29]. A total of 90 animals were used in the study. Rats were randomly allocated to one of three *c-fos* ASO treatment groups: (1) In the ASO group, rats were intrathecally administered *c-fos* ASO (5’‐GAA CAT CAT GGT CGT‐3’) at a dose of 5 μg/10 μL (dissolved in 5% DMSO); (2) rats in the SO group received intrathecal administration of *c-fos* sense oligodeoxynucleotide (SO, 5’‐ACG ACC ATG ATG TTC‐3’), dissolved in 5% DMSO at a dose of 5 μg/10 μL; (3) rats in the DMSO group received intrathecal administration of 5% DMSO (10 μL). Fluorocitrate (at a concentration of 5 μg/10 μL, dissolved in 5% DMSO, Sigma, St. Louis, MO, USA), a potent inhibitor of astrocytes activation, was administered intrathecally. SP600125 (at a concentration of 5 μg/10 μL, dissolved in 5% DMSO, Sigma), a potent inhibitor of JNK activation, was administered intrathecally. IL‐1ra (at a concentration of 5 μg/10 μL, dissolved in 5% DMSO, Sigma), a potent inhibitor of IL‐1RI, was administered intrathecally. 5% DMSO (10 μL) was used in the above groups as a vehicle control. The drugs or DMSO were administrated on a daily basis between the 2nd and 8th day (inclusive) prior to CFA administration (performed on Day 8, Figure 1). There were 30 rats in each group. We used 10 rats for behavioral tests, six rats for immunohistochemical staining, seven rats for Western blot analysis, and seven rats for enzyme‐linked immunosorbent assay (ELISA). Intrathecal drug administration was performed as previously described [2].

### 2.3. Behavioral Tests

Von Frey filaments were applied to test the paw withdrawal threshold (PWT). Briefly, one of a series of Von Frey filaments with gradually increasing stiffness (2, 4, 6, 8, 10, 15, and 26 g) was applied to touch the plantar surface for 5–6 s. Each filament was used 10 times, and a 5‐min interval was left between the different forces. The minimal force that caused acute withdrawal, biting, licking, or shaking of the ipsilateral hind limb and vocalization responses at least five times was considered as the PWT.

### 2.4. Immunohistochemical Staining

The rats were sacrificed on Day 9 to do immunohistochemical staining in spinal cords. The rats were perfused transcardially with 0.01 M phosphate‐buffered saline (150 mL, PBS, pH 7.4), followed by administration of 4% paraformaldehyde in 0.1 M phosphate buffer (500 mL, PB, pH 7.4). The L5 spinal segment was removed, postfixed, and transferred to 25% sucrose (w/v) for cryoprotection. The L5 was cut into transverse sections (30 μm thick) with a freezing microtome (Kryostat 1720; Leitz, Mannheim, Germany) and were subsequently collected in dishes. Sections from the different dishes were subsequently incubated at room temperature overnight with one of the following antibodies (Table 1): (1) goat‐anti GFAP antiserum (ab53554; Abcam, Cambridge, MA); (2) rabbit‐anti Fos antiserum (ab190289; Abcam); (3) rabbit‐anti Fos antiserum (Abcam) and goat‐anti GFAP antiserum (Abcam); (4) rabbit‐anti Fos antiserum (Abcam) and mouse‐anti NeuN antiserum (ab104224; Abcam); (5) goat‐anti GFAP antiserum (Abcam) and rabbit‐anti p‐JNK antiserum (ab219584; Abcam); (6) goat‐anti GFAP antiserum (Abcam) and mouse‐anti IL‐1β antiserum (ab239517; Abcam); (7) goat‐anti Iba‐1 antiserum (ab5076; Abcam) and mouse‐anti IL‐1β antiserum (Abcam); (8) rabbit‐anti NeuN antiserum (ab177487; Abcam) and mouse‐anti IL‐1β antiserum (Abcam); (9) rabbit‐anti NMDAR2B (NR2B) antiserum (ab65783; Abcam) and mouse‐anti NeuN antiserum (Abcam); (10) mouse‐anti NMDAR2B (NR2B) antiserum (ab93610; Abcam) and rabbit‐anti IL‐1RI antiserum (ab244859; Abcam). The antibodies were diluted to their working concentrations in 0.01 M PBS containing 5% (v/v) FBS, 0.3% (v/v) Triton X‐100, 0.05% (w/v) NaN_3_ and 0.25% (w/v) carrageenan (PBS–FBS, pH 7.4). The sections were in 0.01 M PBS for three times, then incubated at room temperature for 2 h in the species‐specific secondary antibodies (in PBS containing 0.3% Triton X‐100): (1) Alexa Fluor 594 conjugated guinea pig anti‐goat IgG (Molecular Probes, Eugene, Oregon); (2) Alexa Fluor 488 conjugated donkey anti‐rabbit IgG (Molecular Probes); (3) Alexa Fluor 488 conjugated donkey anti‐rabbit IgG (Molecular Probes) and Alexa Fluor 594 conjugated guinea pig anti‐goat IgG (Molecular Probes); (4) Alexa Fluor 488 conjugated donkey anti‐rabbit IgG (Molecular Probes) and Alexa Fluor 594 conjugated guinea pig anti‐mouse IgG (1:400 dilution; Molecular Probes); (5) Alexa Fluor 488 conjugated donkey anti‐goat IgG (‐Molecular Probes) and Alexa Fluor 594 conjugated guinea pig anti‐rabbit IgG (Molecular Probes); (6),(7) Alexa Fluor 488 conjugated donkey anti‐goat IgG (Molecular Probes) and Alexa Fluor 594 conjugated guinea pig anti‐mouse IgG (Molecular Probes); (8),(9)Alexa Fluor 488 conjugated donkey anti‐rabbit IgG (Molecular Probes) and Alexa Fluor 594 conjugated guinea pig anti‐mouse IgG (Molecular Probes); (10) Alexa Fluor 488 conjugated donkey anti‐mouse IgG (Molecular Probes) and Alexa Fluor 594 conjugated guinea pig anti‐rabbit IgG (Molecular Probes). The above sections were mounted onto gelatin‐coated glass slides and cover‐slipped with a mixture of 5% (v/v) glycerin and 2.5% (w/v) triethylenediamine in 0.1M PBS after. The sections were viewed with a confocal laser‐scanning microscope (CLSM, Fluoview 1000, Olympus, Tokyo, Japan) with appropriate emission filters for Alexa 594 (593–615 nm) and Alexa 488 (510–525 nm). Digital images were acquired with FLUOVIEW software (Olympus, Tokyo, Japan). We used the Threshold Image function in Measure of MetaMorph 6.1 to measure the immunofluorescent intensity of GFAP immunopositive somata. Five randomly selected sections (Fos and NeuN) of 30‐μm thickness from each rat (*n* = 6 rats; total 30 sections) were counted the number of immunopositive cells and corrected using Abercrombie’s equation: number of neuron = number of neurons counted × *T*/(*T* + *h*) (*T* = thickness of the sections, *h* = the mean diameter of the nuclei of the large or small neurons. The first specific rabbit, goat and mouse primary antibodies were replaced by a mixture of normal goat, rabbit, or mouse sera. We used the above staining methodology for negative control. Immunopositive staining was not viewed.

**Table 1:** Antiserum used in each experimental group for light microscopy.

Purposes	Primary antiserum	Secondary antiserum
GFAP	Goat‐anti GFAP (1:1000)	Alexa fluor 594 conjugated guinea pig anti‐goat (1:400)
Fos	Rabbit‐anti Fos (1:1000)	Alexa fluor 488 conjugated donkey anti‐rabbit (1:400)
Fos/GFAP	Rabbit‐anti Fos (1:1000) Goat‐anti GFAP (1:1000)	Alexa fluor 488 conjugated donkey anti‐rabbit (1:400) Alexa fluor 594 conjugated guinea pig anti‐goat (1:400)
Fos/NeuN	Rabbit‐anti Fos (1:1000) Mouse‐anti NeuN (1:3000)	Alexa fluor 488 conjugated donkey anti‐rabbit (1:400) Alexa fluor 594 conjugated guinea pig anti‐mouse (1:400)
GFAP/p‐JNK	Goat‐anti GFAP (1:1000) Rabbit‐anti p‐JNK (1:1000)	Alexa fluor 488 conjugated donkey anti‐goat (1:400) Alexa fluor 594 conjugated guinea pig anti‐ rabbit (1 :400)
GFAP/IL‐1β	Goat‐anti GFAP (1:1000) Mouse‐anti IL‐1β (1:1000)	Alexa fluor 488 conjugated donkey anti‐goat (1:400) Alexa fluor 594 conjugated guinea pig anti‐ mouse (1:400)
Iba‐1/IL‐1β	Goat‐anti GFAP (1:1000) Mouse‐anti IL‐1β (1:1000)	Alexa fluor 488 conjugated donkey anti‐goat (1:400) Alexa fluor 594 conjugated guinea pig anti‐mouse (1:400)
NeuN/IL‐1β	Rabbit ‐anti NeuN (1:3000) Mouse‐anti IL‐1β (1:1000)	Alexa fluor 488 conjugated donkey anti‐rabbit IgG (1:400) Alexa fluor 594 conjugated guinea pig anti‐ mouse IgG (1:400)
NR2B/NeuN	Rabbit‐anti NR2B (1:1000) Mouse‐anti NeuN (1:3000)	Alexa fluor 488 conjugated donkey anti‐rabbit (1:400) Alexa fluor 594 conjugated guinea pig anti‐ mouse (1:400)
NR2B/IL‐1RI	Mouse‐anti NR2B (1:1000) Rabbit‐anti IL‐1RI (1:1000)	Alexa fluor 488 conjugated donkey anti‐mouse (1:400) Alexa fluor 594 conjugated guinea pig anti‐rabbit (1:400)

### 2.5. Western Blot Analysis

The rats were anesthetized with sodium pentobarbital (60 mg/kg) in 0.9% (w/v) saline. They were killed by decapitation and the protein (50 μg) of the ipsilateral dorsal part of L5 spinal horn of rat was extracted. Samples were sonicated in ice‐cold lysis buffer (50 mM Tris, pH 7.4, 150 mM NaCl, 5 mM EGTA, 0.5% NP‐40, 10 mM NaF, and 1 mM PMSF). After lysis at 4°C for 1 hr, the cell lysates were centrifuged at 12,000 r/min for 5 min. We added SDS buffer to protein extracts to make the protein denature and subject to SDS‐polyacrylamide gelelectrophoresis. The method was used to transfer the protein onto nitrocellulose membranes (Bio‐Rad). After blocked with 5% skimmed milk in TBST (25 mM Tris, pH 7.4, 137 mM NaCl, 2.7 mM KCl, and 0.05% Tween 20) at room temperature for 2 h, the membranes were incubated with primary antibody goat‐anti‐GFAP (1:1000; Abcam) or mouse‐anti‐NR2B (1:1000; Abcam) at 4°C overnight. The membranes were incubated with horseradish peroxidase‐conjugated secondary antibodies (anti‐goat 1:3000, anti‐mouse 1:5000, anti‐rabbit 1:3000; Amersham Pharmacia Biotech Inc., Piscataway, NJ, USA). Between each step, the membranes were washed with Tris‐buffered saline containing 0.02% Tween‐20 (TBST). All reactions were observed by the enhanced chemiluminescence (ECL) detection method (Amersham). The results of chemiluminescent densities from the protein bands were normalized to β‐actin levels (loading control), using Labworks Software (Ultra‐Violet Products, UK).

### 2.6. ELISA

We used ELISA to determine the concentrations of IL‐1β in the spinal cord at Day 9. The animals were killed, and the lumbar segments of the spinal cord were sonicated. The protein was centrifuged at 3000 r/min for 15 min. IL‐1β protein was estimated by ELISA kits according to the manufacturer’s protocol (RapidBio Lab, Calabasas, CA).

### 2.7. Statistical Analysis

Data were expressed as means ± S.E.M. *p*  < 0.05 was considered as statistically significant. One‐way analysis of variance (ANOVA) across groups was used to compare the immunofluorescent intensity of GFAP, the number of Fos‐positive or NeuN‐positive cells, and the protein levels of GFAP and NR2B. ELISA and mechanical allodynia data were analyzed using one‐way ANOVA (groups by days). Students‐Newman–Keuls method was used as a post hoc test to detect differences between groups.

## 3. Results

### 3.1. SPS + CFA Treatment Significantly Activated Both Neurons and Astrocytes in the Spinal Dorsal Horn

In order to examine whether SPS, CFA, and SPS + CFA induced neuron and astrocyte activation in the spinal dorsal horn, we used GFAP and Fos immunostaining. The activatedastrocyte was seen after SPS, CFA, and SPS + CFA in the ipsilateral but not the contralateral dorsal horn (*p*  < 0.05). The activated astrocyte presented hypertrophy (Figure 2A,B,C). There were significant differences between groups in relation to the fluorescence intensity of GFAP by one‐way ANOVA (*F*_3,12_ = 68.63, *p*  < 0.001). The intensity of GFAP immunoreactivity was significantly increased in SPS or CFA or SPS + CFA compared with naïve (*p*  < 0.05). Increased intensity of GFAP immunoreactivity was found in SPS + CFA compared with CFA or SPS (*p*  < 0.05, Figure 2D,E). The expression of Fos was significantly increased in SPS + CFA compared with CFA or SPS in the ipsilateral but not the contralateral dorsal horn (*p*  < 0.05, Figure 3A–D). The vast majority of Fos‐immunoreactive (Fos‐IR) cells coexpressed NeuN, confirming their neuronal identity (Figure 3E,F). Double immunohistochemistry staining disclosed that GFAP and Fos immunoreactivity in the superficial laminae of the dorsal horn ipsilateral, and in the superficial laminae of the dorsal horn, GFAP‐IR processes are distributed around Fos‐IR neurons on the ipsilateral side. (Figure 3G,H). There were significant differences in GFAP protein levels in the spinal dorsal horn between groups by one‐way ANOVA (*F*_3, 12_ = 117.57, *p*  < 0.001). Western blot analysis revealed significantly increased GFAP expression in the SPS‐, CFA‐, and SPS + CFA‐treated rats compared to naïve controls (*p*  < 0.05). Furthermore, GFAP levels were significantly higher in the SPS + CFA group than in the CFA‐only group or SPS‐only group (*p*  < 0.05) (Figure 4A,B).

Figure 2Immunofluorescent labeling of GFAP (glial fibrillary acidic protein) in the spinal dorsal horn following SPS + CFA treatment. (A,C) Immunofluorescent labeling revealed a significant increase in GFAP expression in the ipsilateral dorsal horn compared to the contralateral side ( ^∗^ *p*  < 0.05). (B) Immunoperoxidase labeling provided an overview of the examined region (laminae I–II). Scale bars: 250 μm (A), 300 μm (B). (D,E) Representative micrographs demonstrated a marked upregulation of GFAP on Day 9 in the SPS + CFA group ( ^∗^ *p*  < 0.05 vs. naïve group; #*p*  < 0.05 vs. CFA group; ^@^ *p*  < 0.05 vs. SPS group). Scale bar: 150 μm (E).

**Figure:**
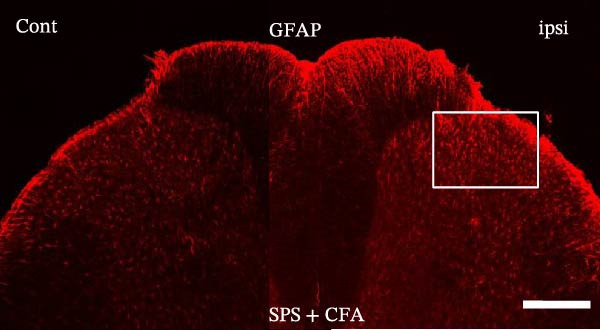
(A)

**Figure:**
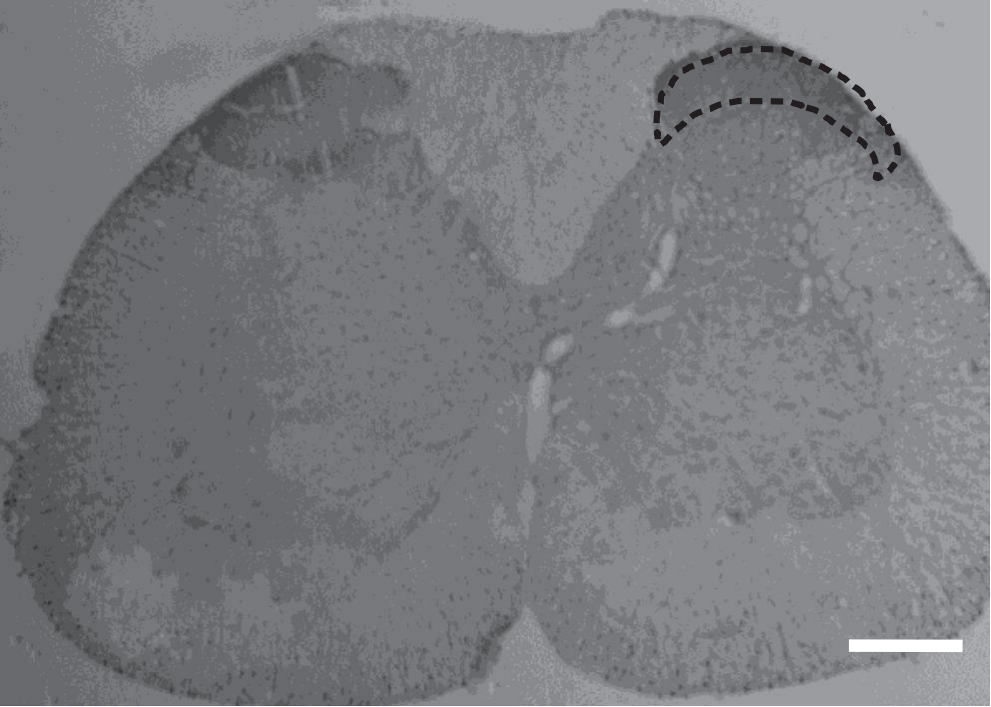
(B)

**Figure:**
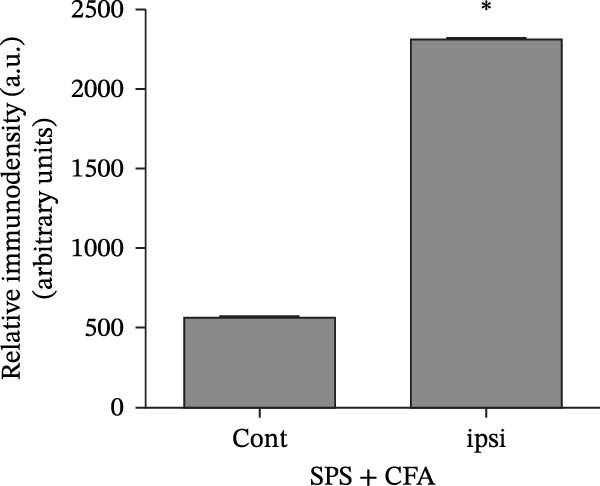
(C)

**Figure:**
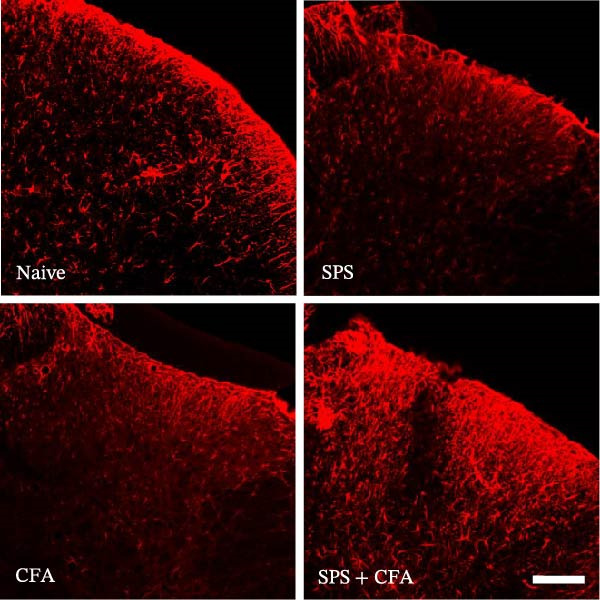
(D)

**Figure:**
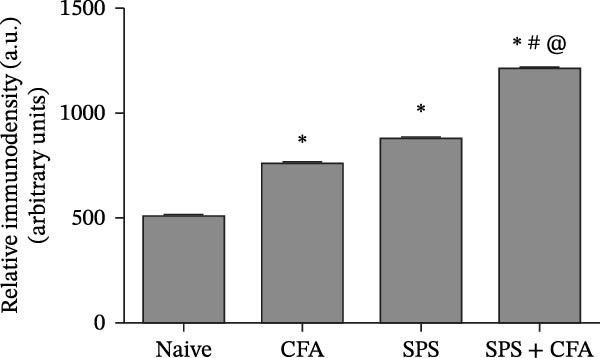
(E)

Figure 3The immunofluorescence labeling of Fos/NeuN and Fos/GFAP in the ipsilateral dorsal horn following SPS + CFA. (A,B) Immunofluorescent labeling revealed a significant increase in Fos expression in the ipsilateral dorsal horn on Day 9 compared to the contralateral side ( ^∗^ *p*  < 0.05). Scale bar: 200 μm (A). (C,D) Fos‐immunoreactive (Fos‐ir) neurons in different treatment groups. The number of Fos‐ir neurons per section in the spinal dorsal horn was quantified ( ^∗^ *p*  < 0.05 vs. naïve group; ^#^ *p*  < 0.05 vs. CFA group; ^◆^ *p*  < 0.05 vs. SPS group). Scale bar: 100 μm (C). (E–I): Colocalization of Fos (green) with GFAP (red) or NeuN (red) was observed in the ipsilateral dorsal horn after SPS + CFA. Enlarged views of boxed regions in (E, G) are shown in (F, H). Quantification revealed the proportion of Fos/NeuN (yellow) double‐labeled neurons among total Fos‐positive cells. Scale bars: 100 μm (E, G); 80 μm (F, H).

**Figure:**
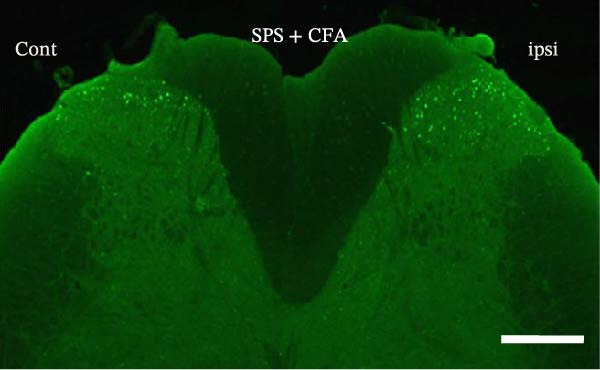
(A)

**Figure:**
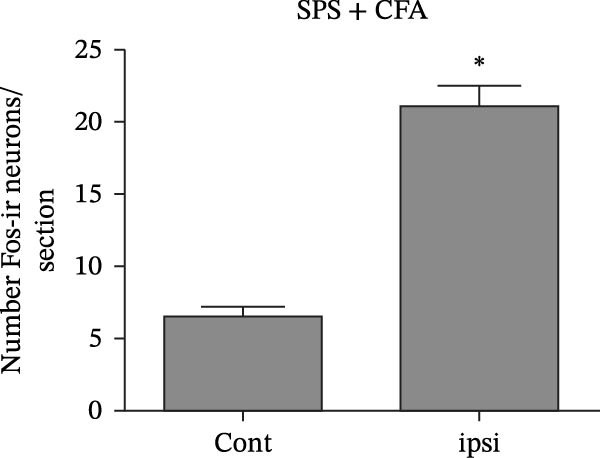
(B)

**Figure:**
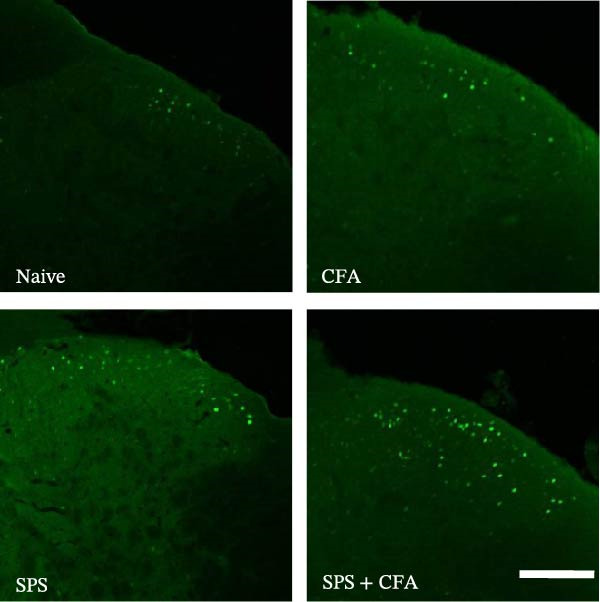
(C)

**Figure:**
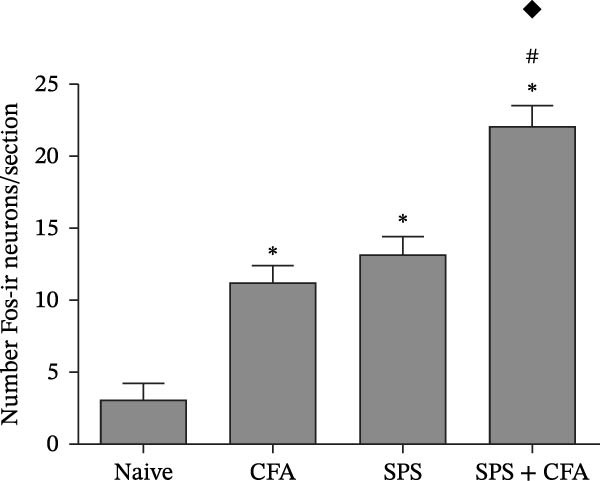
(D)

**Figure:**
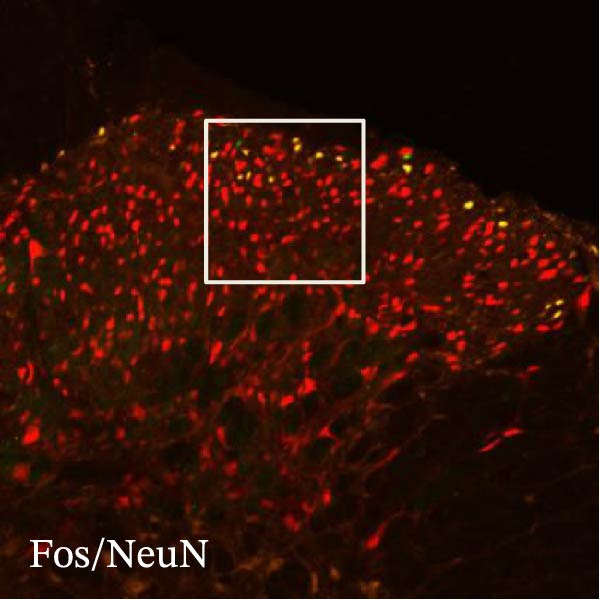
(E)

**Figure:**
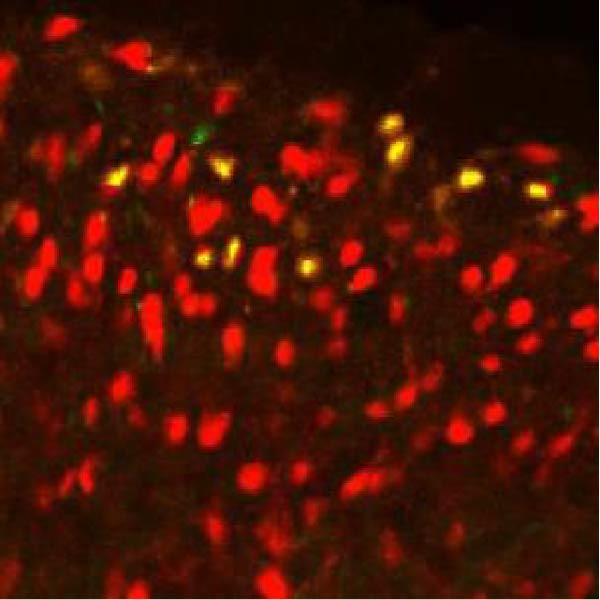
(F)

**Figure:**
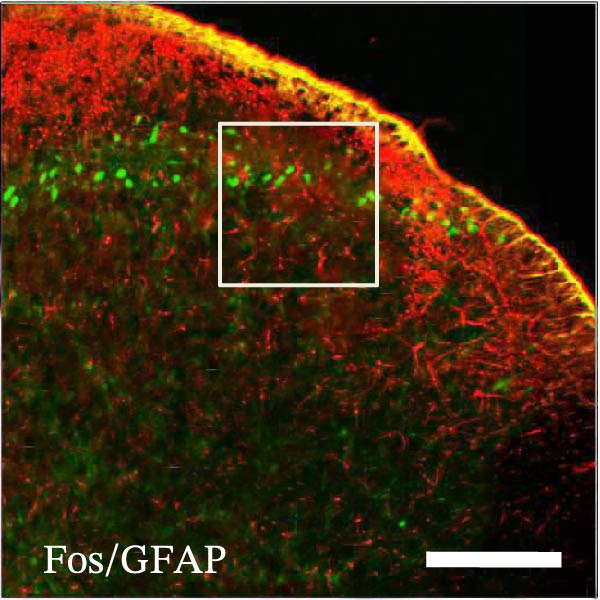
(G)

**Figure:**
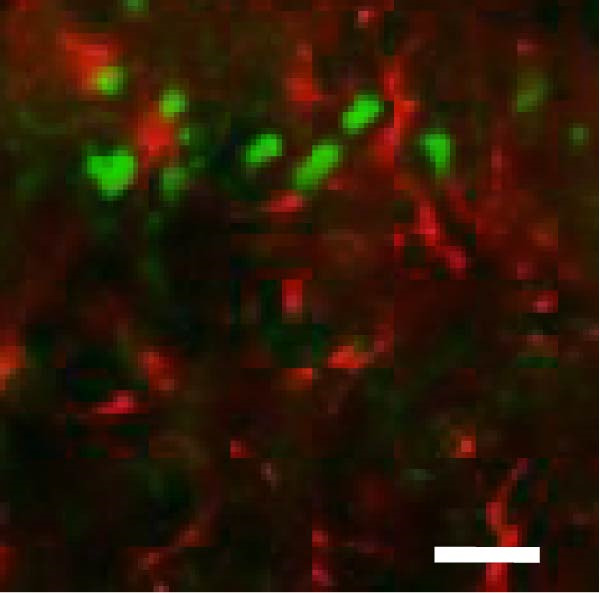
(H)

**Figure:**
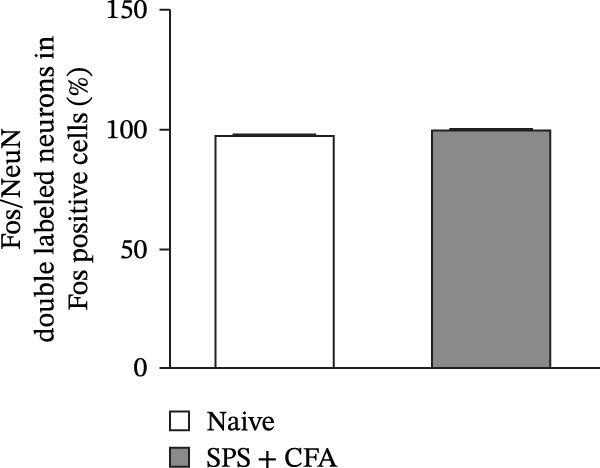
(I)

Figure 4(A) Immunoblots of GFAP in the spinal dorsal horn across treatment groups (50 μg total protein per lane). (B) Densitometric analysis revealed a significant upregulation of GFAP protein levels in the SPS, CFA, and SPS + CFA groups compared to naïve controls ( ^∗^ *p*  < 0.05). Moreover, GFAP expression was further elevated in the SPS + CFA group relative to either CFA or SPS group alone (^#^ *p*  < 0.05 vs. CFA group; ^@^ *p*  < 0.05 vs. SPS group).

**Figure:**
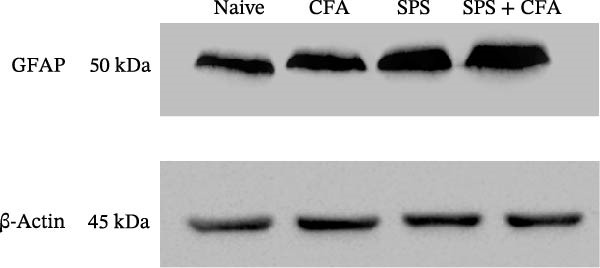
(A)

**Figure:**
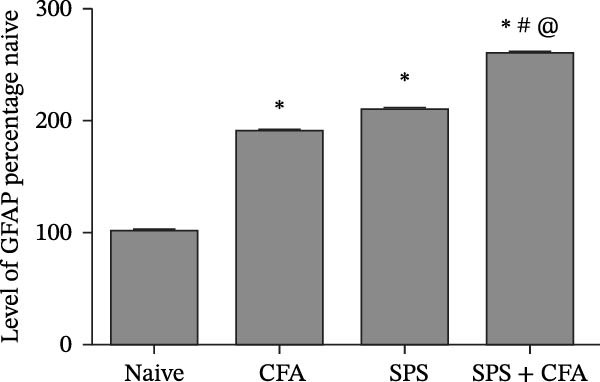
(B)

### 3.2. The CFA + SPS Model Induced Significant Activation of Astrocytic JNK Signaling in the Spinal Dorsal Horn

JNK activation (JNK phosphorylation) was revealed by immunohistochemistry in the superficial laminae of the dorsal horn ipsilateral after SPS + CFA. There were significant differences in pJNK levels in the spinal dorsal horn between groups by one‐way ANOVA (*F*_3, 12_ = 74.23, *p*  < 0.001). The expression of pJNK levels was significantly increased in SPS + CFA compared with CFA or SPS in the ipsilateral but not the contralateral dorsal horn (*p*  < 0.05). The double immunofluorescence method showed that pJNK was totally colocalized with GFAP, not with NeuN or Iba‐1 (Figure 5).

Figure 5The effect of SPS + CFA on phosphorylated JNK (p‐JNK) activation. Double immunofluorescence showed that GFAP colocalized completely with p‐JNK in the ipsilateral but not contralateral spinal dorsal horn. An area of (G,H) is enlarged in (B,F), respectively. Scale bars = 200 μm in (A–F) and 70 μm in (G–I).

**Figure:**
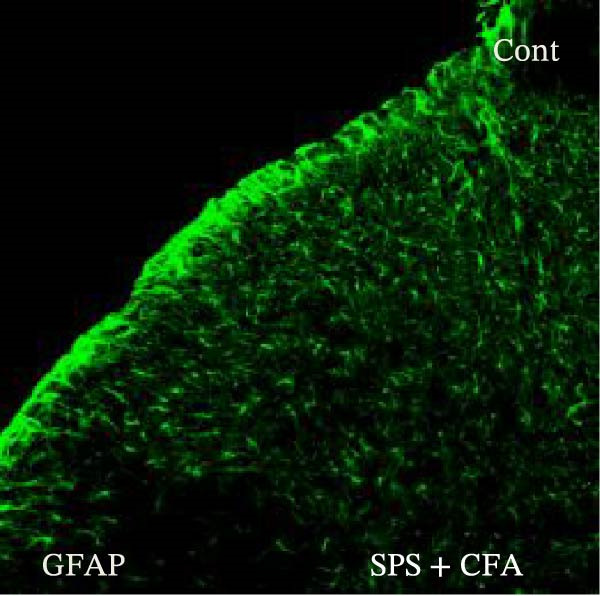
(A)

**Figure:**
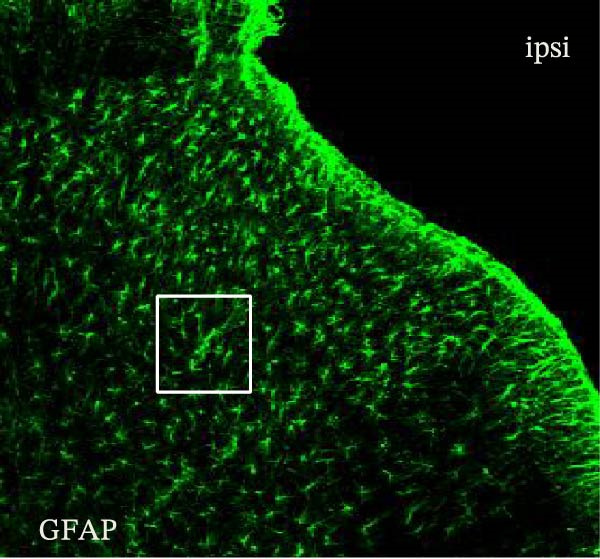
(B)

**Figure:**
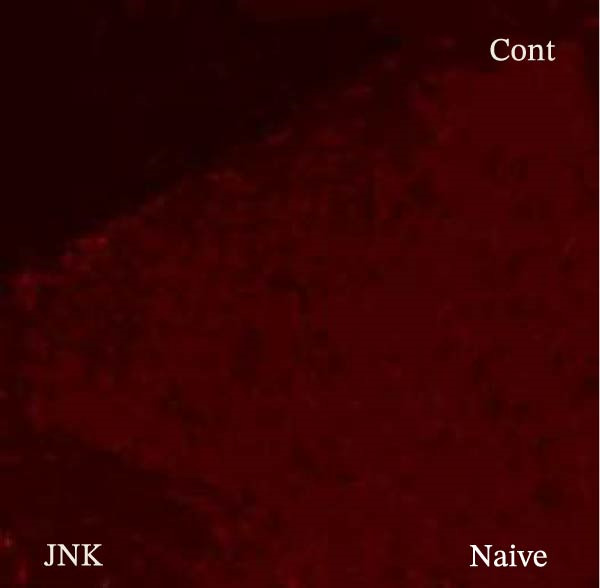
(C)

**Figure:**
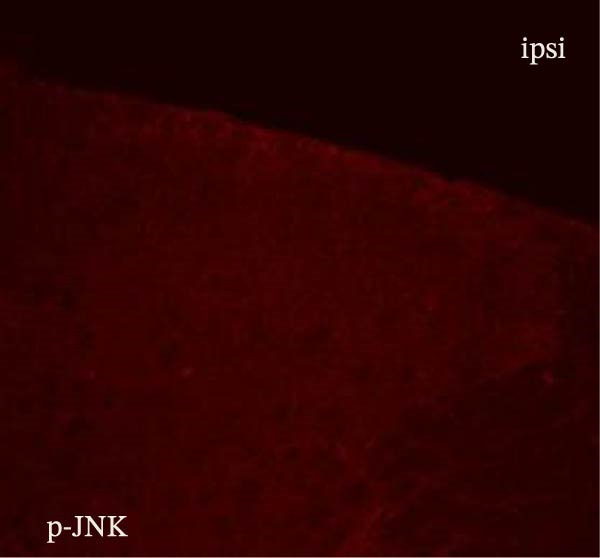
(D)

**Figure:**
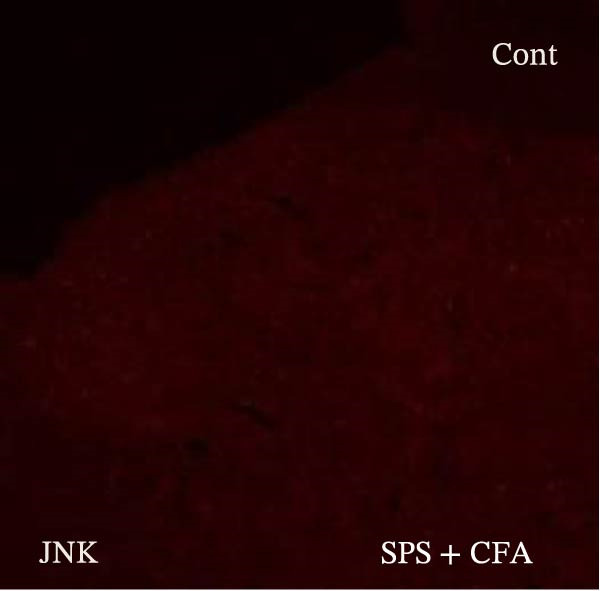
(E)

**Figure:**
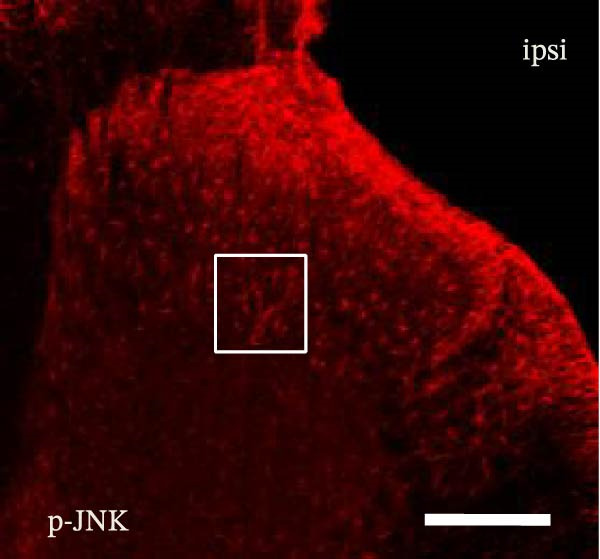
(F)

**Figure:**
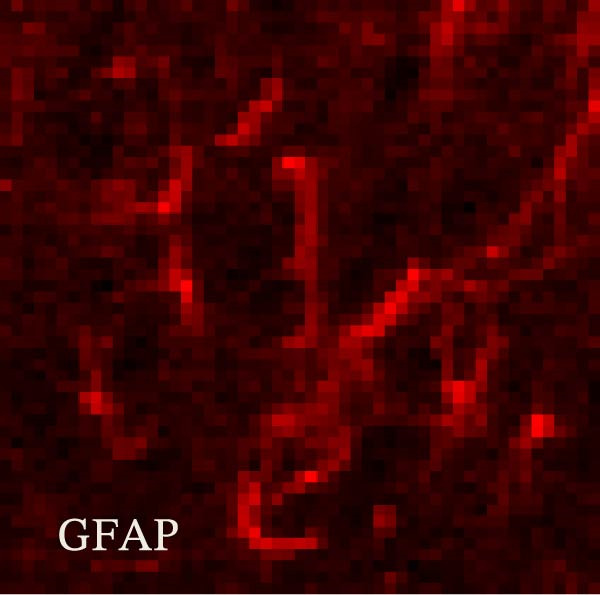
(G)

**Figure:**
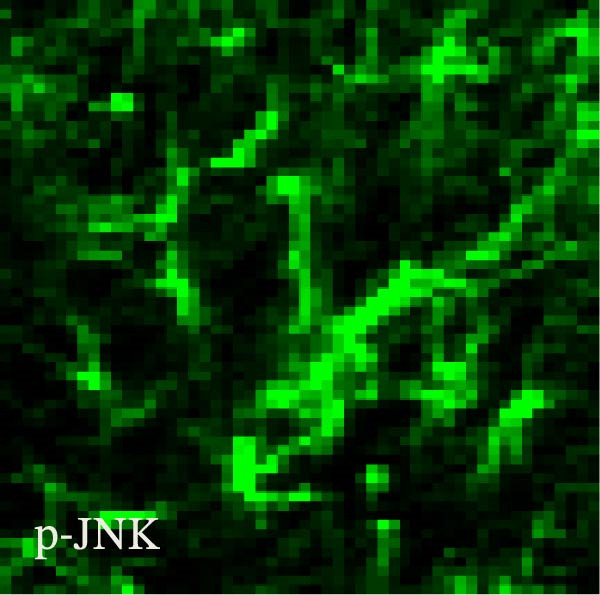
(H)

**Figure:**
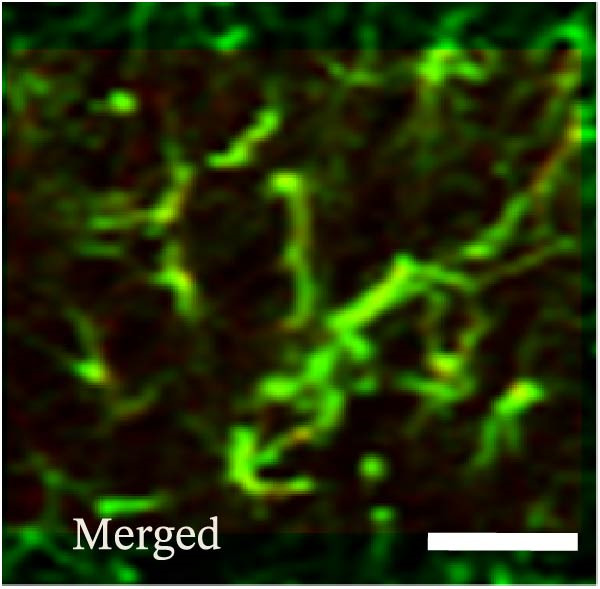
(I)

### 3.3. The CFA + SPS Model Significantly Upregulated IL−1β Expression in Spinal Astrocytes

**Figure 6:**
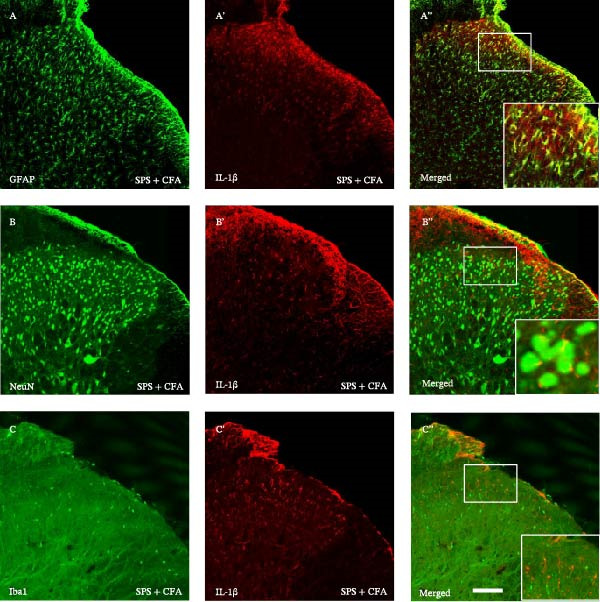
Double labeled IL‐1β with GFAP (A–A”), NeuN (B–B”) or Iba‐1 (C–C”). A–A” show double labeling of IL‐1β with GFAP, a marker of astrocytes. (B–B”) show lack double labeling of IL‐1β with NeuN, a marker of neurons. (C–C”) show lack double labeling of IL‐1β with Iba‐1, a marker of microglia. Examples of double‐labeled neurons are indicated in the bottom of (A”,B”,C”). Scale bars = 200 μm.

**Figure 7:**
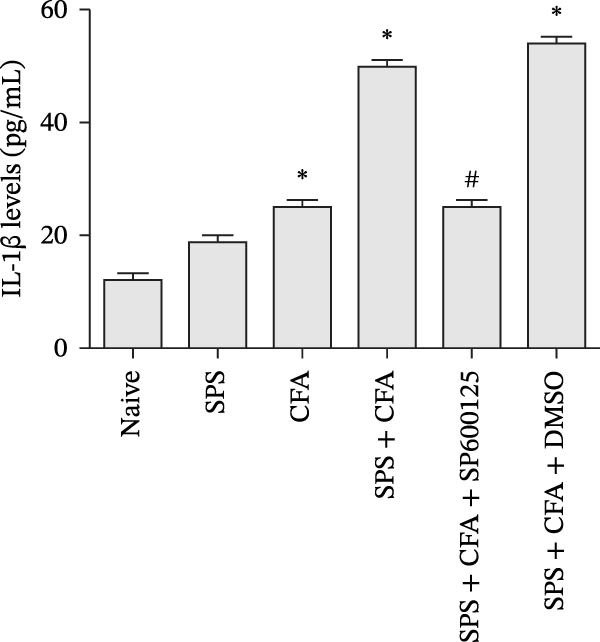
The effect of CFA + SPS and chronic SP600125 administration on IL‐1β production by ELISA. An increase in IL‐1β production was shown after CFA + SPS. SP600125 administration reduces the CFA + SPS‐induced increase in IL‐1β production ( ^∗^ *p*  < 0.05 vs. the naïve group; # *p*  < 0.05 vs. the SPS + CFA group).

In order to determine whether activation of astrocytes by SPS + CFA with an increase in inflammatory cytokine levels, we used double immunohistochemistry staining to indicate that IL‐1β was collocated with GFAP, but not with NeuN or Iba‐1 in the superficial laminae of the dorsal horn ipsilateral after SPS + CFA (Figure 6). Furthermore, we observed CFA + SPS on IL‐1β production by ELISA. There were significant differences in IL‐1β production in the spinal dorsal horn between groups by one‐way ANOVA (*F*_3, 12_ = 92.18, *p*  < 0.001). An increase in IL‐1β production was observed after CFA + SPS compared to the CFA or naïve group (*p*  < 0.05). Intrathecal injection of SP600125, a specific JNK inhibitor, could reduce the CFA + SPS‐induced increase in IL‐1β production (Figure 7).

### 3.4. IL−1β Signaling Potentiates NR2B‐Containing NMDAR Activation in the CFA + SPS Model

Our above results showed that Fos‐IR neurons were close with the GFAP‐IR processes. To study the interaction of astrocyte activation and neuronal activity, we examined NMDAR activation after SPS + CFA. Double immunostaining showed that NR2B‐containing NMDAR colocalized with NeuN by SPS + CFA in the ipsilateral but not the contralateral dorsal horn neurons (Figure 8). Immunostaining revealed coexpression of IL‐1 receptor (IL‐1RI) and NR2B specifically in ipsilateral dorsal horn neurons after SPS + CFA administration (Figure 9). There were significant differences in IL‐1β production in the spinal dorsal horn between groups by one‐way ANOVA (*F*_3, 12_ = 124.63, *p*  < 0.001). There was also upregulation of NR2B‐containing NMDAR protein levels in SPS + CFA groups compared with CFA or SPS group (*p*  < 0.05). Treatment with IL‐1RI antagonist IL‐1ra blocked the increase in NR2B‐containing NMDAR protein levels in SPS + CFA groups (Figure 10). These results propose a mechanistic link where IL‐1β‐IL‐1R signaling gates NR2B‐NMDAR activation.

Figure 8Localization of NR2B in the ipsilateral but not contralateral spinal dorsal horn after CFA + SPS. (A) NR2B is shown as green fluorescence (Alexa Fluor 488). (B) NeuN is visualized with (Alexa Fluor 594). (C, D) Double immunofluorescence labeling with NeuN (nucleus) and NR2B (cytoplasm) in the same neurons. An area of C is enlarged in (D). Examples of double‐labeled neurons are indicated in the bottom of D. Scale bars = 200 μm in (A,B,C) and 70 μm in (D).

**Figure:**
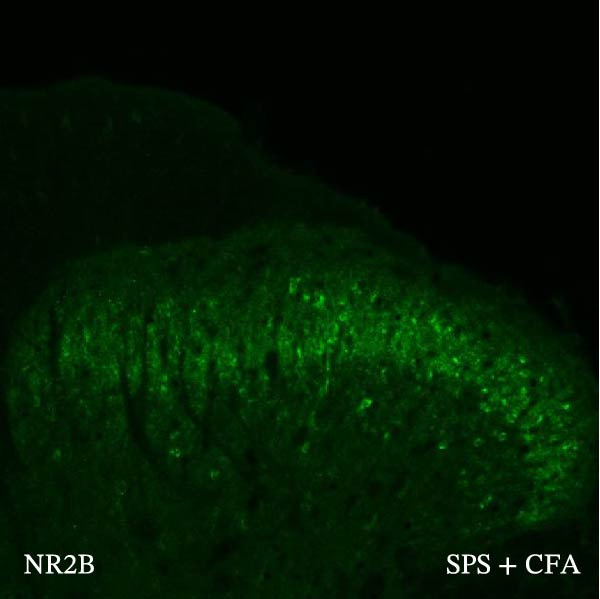
(A)

**Figure:**
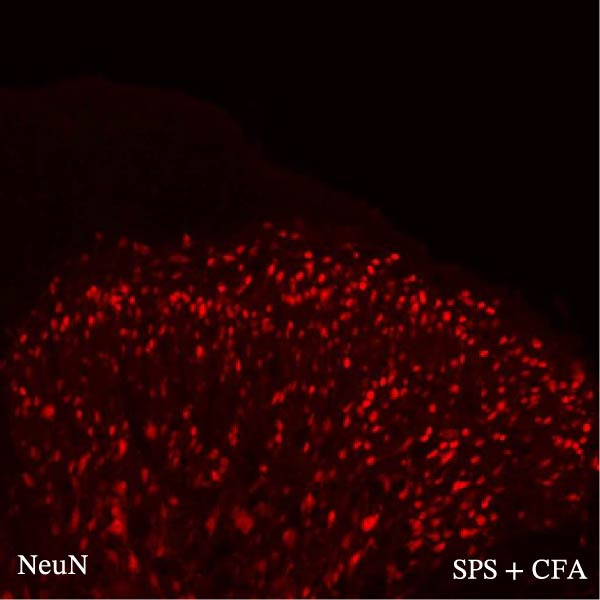
(B)

**Figure:**
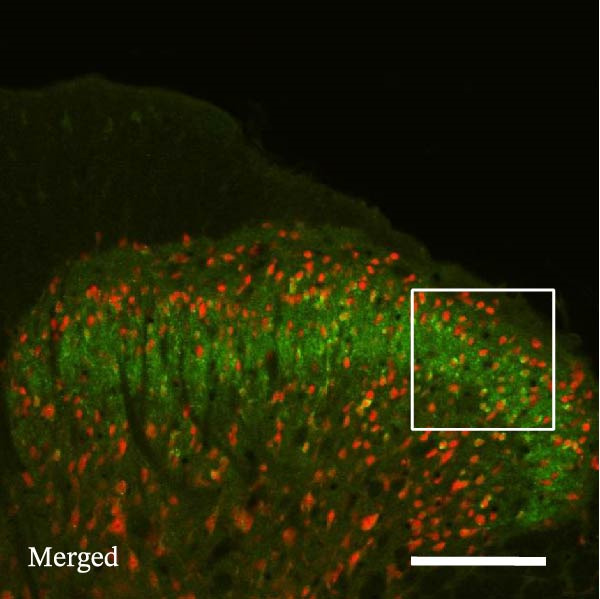
(C)

**Figure:**
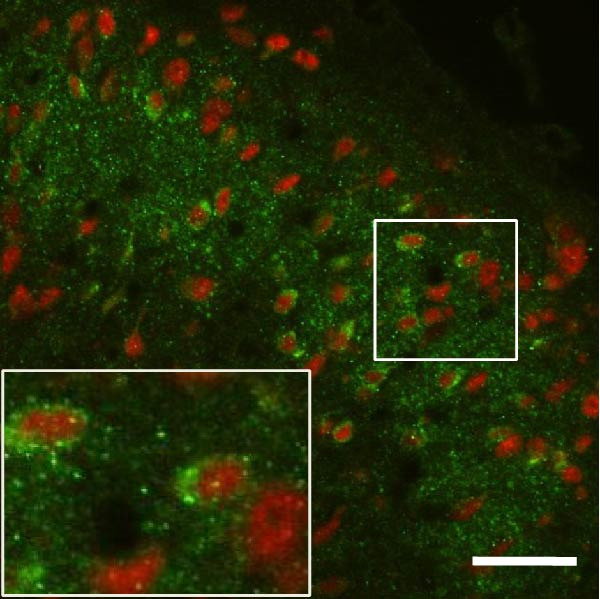
(D)

Figure 9Colocalization of IL‐1RI with NR2B in the ipsilateral but not contralateral spinal dorsal horn after CFA + SPS. (A) NR2B is visualized with Alexa Fluor 488. (B) IL‐1RI is shown as Alexa Fluor 594. (C) Overlap of left and middle panels reveals neurons that exhibit double fluorescence, suggesting colocalization of IL‐1RI and NR2B. Scale bars = 200 μm. (D) Enlarged views of boxed regions in (C), Scale bars = 80 μm.

**Figure:**
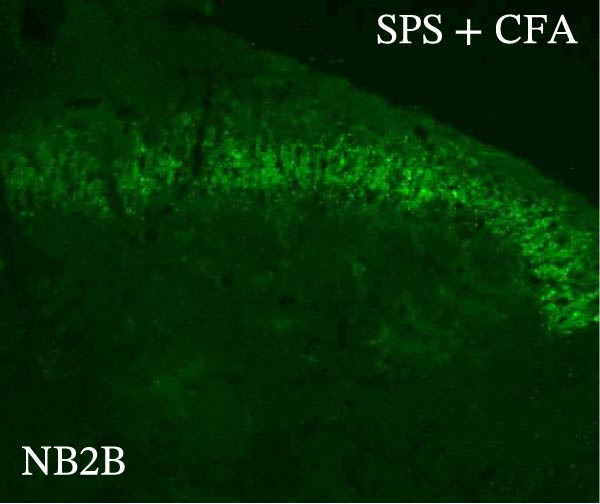
(A)

**Figure:**
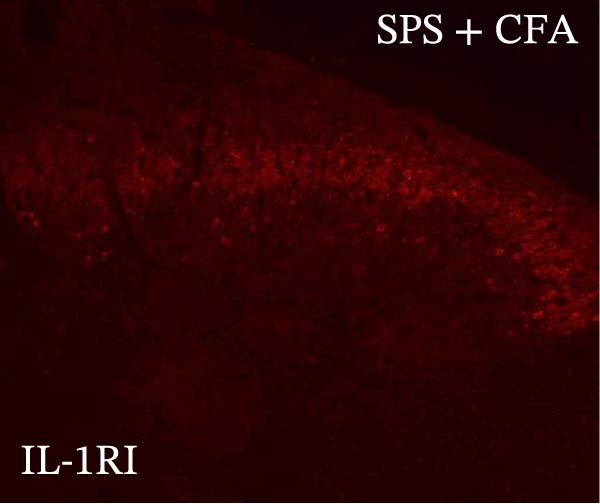
(B)

**Figure:**
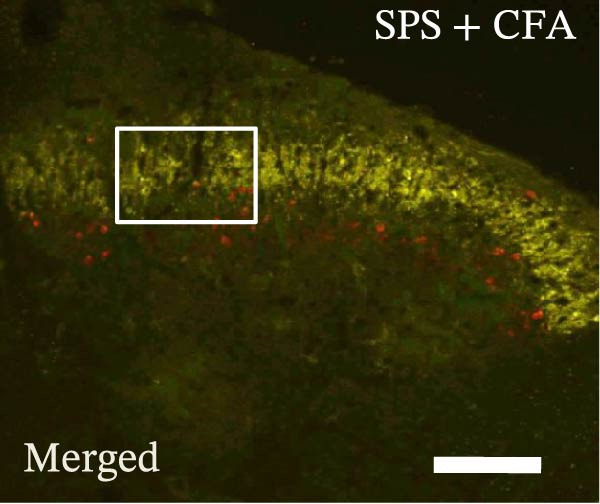
(C)

**Figure:**
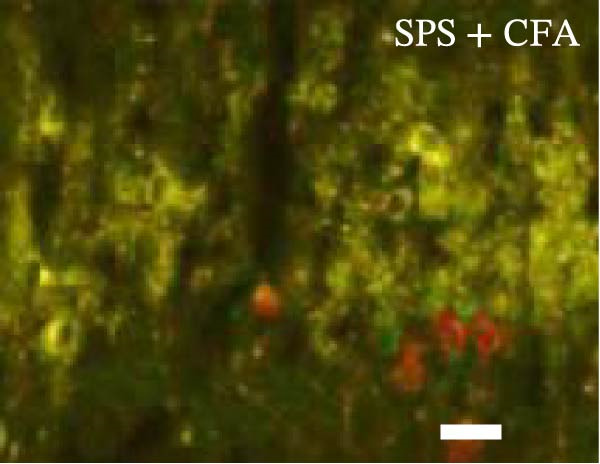
(D)

Figure 10Immunoblots of NR2B in the spinal dorsal horn across treatment groups (50 μg total protein per lane) (A). (B) Densitometric analysis revealed a significant upregulation of NR2B protein levels in the CFA, and SPS + CFA groups compared to naïve controls (*p*  < 0.05). Moreover, GFAP expression was further elevated in the SPS + CFA group relative to either CFA or SPS group alone. Treatment with IL‐1RI antagonist IL‐1ra blocked the increase in NR2B protein levels ( ^∗^ *p*  < 0.05 vs. naïve group; ^#^ *p*  < 0.05 vs. CFA group; ^@^ *p*  < 0.05 vs. SPS group; ^&^ *p*  < 0.05 vs. SPS + CFA group).

**Figure:**
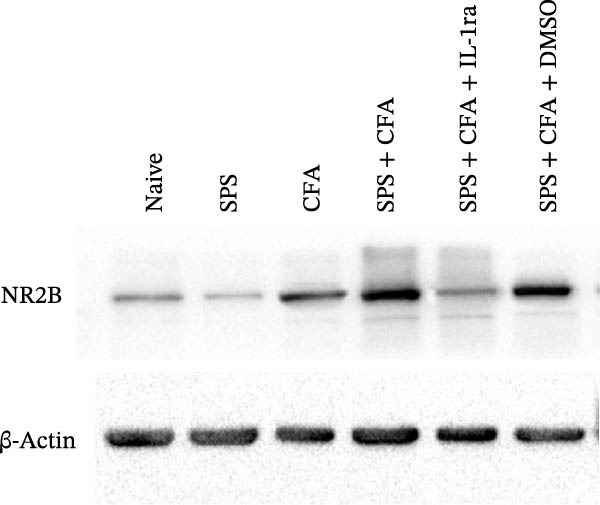
(A)

**Figure:**
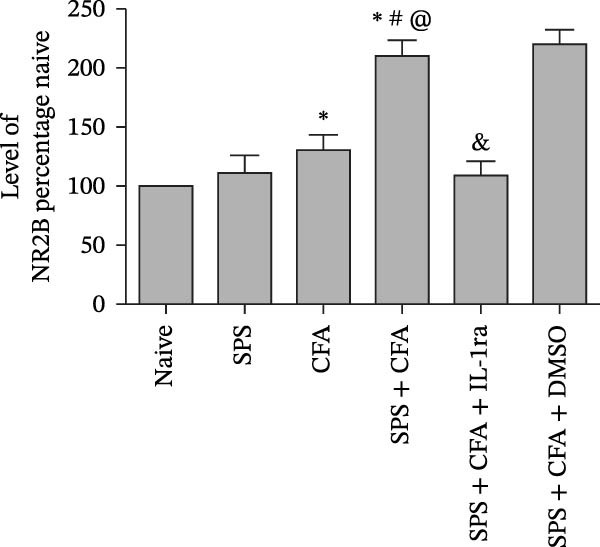
(B)

### 3.5. Effect of c‐fos ASO or Fluorocitrate or IL−1ra or SP600125 on Mechanical Allodynia in Rat After SPS + CFA Treatment

**Figure 11:**
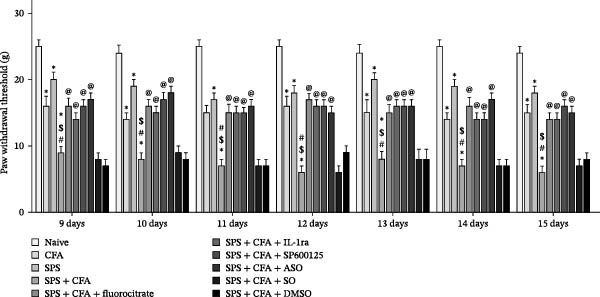
Comparison of CFA + SPS and different administration in relation to mechanical hyperalgesia. SPS + CFA‐exposed rats had significantly induced mechanical hyperalgesia as shown by the Von Frey tests. The paw withdrawal threshold (PWT) was reduced in the injured hindpaw of the SPS + CFA‐exposed rats compared with the naïve rats. Chronic treatment with Fluorocitrate significantly attenuated allodynia in SPS + CFA‐exposed rats (*p*  < 0.05). Chronic treatment with c‐fos ASO significantly attenuated allodynia in SPS + CFA‐exposed rats (*p*  < 0.05). In addition, no significant difference existed between the DMSO treatment group and the SO group (*p*  > 0.05). IL‐1RI antagonist IL‐1ra significantly attenuated behavioral hyperalgesia induced by SPS + CFA (*p*  < 0.05). JNK antagonist SP600125 significantly attenuated behavioral hyperalgesia induced by SPS + CFA (*p*  < 0.05,  ^∗^ *p*  < 0.05 vs. naïve group; ^#^ *p*  < 0.05 vs. CFA group; ^$^ *p*  < 0.05 vs. SPS group; @ *p*  < 0.05 vs. SPS + CFA group).

The mechanical allodynia developed after SPS, CFA, SPS + CFA was assessed by applying a series of von Frey microfilaments to the skin of the right hindpaw. The response reflex to a range of von Frey filament forces was determined. These measurements reached a lower PWT in the von Frey in the SPS group from Day 7 (*p*  < 0.05), the CFA group after CFA injection (*p*  < 0.05), and the SPS + CFA‐exposed group (*p*  < 0.01) from Day 7 compared to the naïve rats in our present and previous analysis. There was a significant effect (*F*_3,132_ = 95.72, *p*  < 0.001) by one‐way ANOVA on the PWTs. Analysis revealed that SPS + CFA‐exposed rats from Day 9 after CFA injection produced a significant decrease in the mechanical withdrawal threshold (MWT) compared to CFA‐exposed rats (*p*  < 0.05). Chronic treatment with fluorocitrate significantly attenuated allodynia in SPS + CFA‐exposed rats (*p*  < 0.05). Chronic treatment with c‐fos ASO significantly attenuated allodynia in SPS + CFA‐exposed rats (*p*  < 0.05). In addition, no significant difference existed between the DMSO treatment group and the SO group (*p*  > 0.05). IL‐1RI antagonist IL‐1ra significantly attenuated behavioral hyperalgesia induced by SPS + CFA (*p*  < 0.05). JNK antagonist SP600125 significantly attenuated behavioral hyperalgesia induced by SPS + CFA (*p*  < 0.05, Figure 11).

## 4. Discussion

Glia consists of oligodendrocytes, microglia, and astrocytes in the central nervous system. Spinal glial activation is viewed as a vital role in the process of allodynia and hyperalgesia in different kinds of chronic pain [30–32]. Our previous studies showed the presence of a “crosstalk” between activated microglia and neurons in the spinal dorsal horn, which might contribute to the stress‐induced hyperactivated state [2]. In the present research, we got the following results: (1) The activation of astrocytes was demonstrated by increased immunoreactivity towards GFAPs (a marker of astroglia) from stressed rats and CFA‐treatment rats. Moreover, a further increase in GFAP activation was observed in the spinal dorsal horn after SPS +CFA compared with SPS or CFA. The activated astrocyte exhibited hypertrophy, and GFAP‐IR processes were in close with Fos‐IR neurons (a marker for activated neurons). (2) We observed that p‐JNK was colocalized with GFAP in the spinal cord after SPS +CFA following immunofluorescence. Our ELISA data revealed that CFA + SPS administration significantly elevated IL‐1β levels. Intrathecal administration of SP600125, a specific JNK inhibitor, attenuated the CFA + SPS‐induced upregulation of IL‐1β. Immunofluorescence analysis further revealed that the inflammatory cytokine IL‐1β was selectively localized to astrocytes following SPS + CFA treatment. (3) Our results demonstrated that NR2B in the spinal cord was activated, as evidenced by upregulated NR2B protein levels in the SPS + CFA group compared to the CFA‐ or SPS‐alone groups. Immunofluorescence analysis revealed that the expression of NR2B, at least based on morphological assessment, indicates its presence on NeuN‐positive neurons following SPS + CFA. Additionally, IL‐1RI was found to colocalize with NR2B in spinal cord neurons after SPS + CFA exposure. Notably, treatment with the IL‐1RI antagonist IL‐1ra abolished the SPS + CFA‐induced increase in NR2B protein expression. (4) Enhanced behavioral mechanical hyperalgesia was observed in SPS + CFA than SPS or CFA. Both chronic administration of either fluorocitrate or c‐fos ASO attenuated the SPS + CFA‐induced hyperalgesia. (5) Inhibition of p‐JNK activation by SP600125 and inhibition of IL‐1RI activation by IL‐1RA attenuated the SPS + CFA‐induced hyperalgesia.

Extensive research demonstrates that psychological stress significantly contributes to the initiation and amplification of pain. Moreover, clinical evidence underscores the role of chronic stress in promoting increased pain sensitivity, a condition described as “SIH” [33, 34]. It is well established that PTSD, a chronic stress and anxiety disorder, leads to an increase in pain sensitivity [35]. However, the underlying cellular and molecular mechanism in the spinal dorsal horn remains to be elucidated. It is well known that stress produces persistently increased levels of hormones, including glucocorticoid hormone [36]. Several data suggest that glia might be a target of glucocorticoid hormone in the central nervous system, these and may respond to different stress conditions [37]. Our previous studies showed that SPS results in microglial activation in the spinal dorsal horn [2]. In the present study, we found that SPS also results in astrocytes activation in the spinal dorsal horn using CFA, SPS, and SPS + CFA combined animal, and SPS + CFA‐treated rats produce significantly higher levels of GFAP activation than CFA‐treated or SPS‐treated rats in the spinal dorsal horn. These results are in agreement with studies demonstrating that stress might affect immune responses, including astrocyte activation within the central nervous system. An increase in GFAP activation may account for the occurrence of SIH.

Glia can become “activated” after damage to peripheral tissues and release chemical mediators that modulate neuronal activity and synaptic strength [38]. Astrocytes are intimately close with neuronal synapses by processes contacted with neurons. Our results showed that the activated astrocyte exhibited hypertrophy, and GFAP‐IR processes were in close with Fos‐IR neurons (a marker for activated neurons) after SPS + CFA. These results might suggest that activated astrocyte may promote heightened pain‐like states via astrocyte–neuron signaling pathways. However, the precise cellular and molecular mechanisms underlying astrocyte–neuron signaling in mediating PTSD‐induced hyperalgesia within the spinal dorsal horn remain poorly understood.

Accumulating evidence demonstrates that both astrocyte and microglial activation drive pro‐inflammatory cascades, which can induce detrimental effects such as neuronal hyperexcitability, neurotoxic signaling, and sustained neuroinflammation. Neuroinflammation plays a key role in the pathophysiological mechanisms underlying neuropathic pain after peripheral nerve injury. It is now well‐established that cytokines, chemokines, and neuromodulators in the CNS, alongside activated glial cells, may regulate or alter neuronal activity. Pro‐inflammatory mediators released from activated astrocytes can act on neuronal functions or glial cells, generating a neuro‐glial amplification loop in chronic restraint stress and pain [30, 39]. Our findings revealed that SPS + CFA exposure increased IL‐1β expression compared to SPS group or CFA group (*p*  < 0.05), with this pro‐inflammatory cytokine predominantly localized within astrocytes. This might suggest astrocytes activation and cytokine releasing might be one of the factors that facilitate SPS‐induced hyperalgesia. Astrocyte activation triggers the phosphorylation and subsequent activation of various MAPK pathways, such as extracellular signal‐regulated kinase (ERK/MAPK) and c‐JNK (MAPK) [40]. This activation cascade ultimately enhances the production of pro‐inflammatory mediators, including IL‐1β, IL‐6, tumor necrosis factor‐α (TNF‐α), prostaglandin E2 (PGE2), and nitric oxide (NO) [41]. In the present study, we observed that p‐JNK was colocalized with GFAP in the spinal cord after SPS + CFA following immunofluorescence. These results demonstrate that chronic stress exacerbates CFA‐triggered astrocyte activation by promoting JNK pathway signaling. Furthermore, we found increased in IL‐1β production was observed after CFA + SPS compared to CFA or naïve group (*p*  < 0.05). Intrathecal injection of SP600125, a specific JNK inhibitor, could reduce the CFA + SPS‐induced increase in IL‐1β production. Behavioral tests revealed that PTSD‐pain animals developed hyperalgesia, which was partially alleviated by administration of fluorocitrate (an astrocyte inhibitor), IL‐1ra (an IL‐1RI antagonist), or SP600125 (a JNK‐specific inhibitor).

However, a formal repeated‐measures comparison was not conducted. Rather, the results showed the effects of c‐fos ASO, fluorocitrate, IL‐1ra, or SP600125 on mechanical allodynia in SPS + CFA‐treated rats. The findings suggest that JNK‐dependent astrocyte activation promotes IL‐1β production, which in turn drives SIH.

Previous studies have shown that NMDARs are involved in persistentpain by synaptic plasticity [30, 42]. Several studies indicated that NMDAR activation in the spinal dorsal horn or the trigeminal transition zone contributed to inflammation pain [26, 43]. However, few reports on whether NMDAR activation in the spinal dorsal horn is associated with SIH. Our results showed that there was upregulation of NMDAR2B (NR2B) protein levels in SPS + CFA‐treated animals compared with CFA‐ or SPS‐treated animals (*p*  < 0.05). The results suggest that NR2B in the spinal dorsal horn was activated following SPS + CFA treatment. In our study, there are many NR2B (green) labeled profiles which are not associated with NeuN labeled neuronal nuclei. However, the contours marked by NB2B are distributed around the NeuN‐positive neurons. It is possible that, at least in terms of morphology, the expression of NR2R indicates its presence on the NeuN‐positive neurons. We also found that NR2B colocalized with NeuN and IL‐1RI, suggesting that there was a close interaction of inflammatory cytokine signaling with neuronal NR2B in SIH. We further showed that treatment with the IL‐1RI antagonist IL‐1ra blocked the increase in NR2B protein levels following SPS + CFA treatment. The results demonstrate that astrocyte‐derived IL‐1β activates neuronal NR2B via IL‐1RI signaling, contributing to SIH. Future investigations should elucidate the detailed signaling cascade underlying IL‐1β/IL‐1RI‐mediated NR2B activation in SPS + CFA‐induced hyperalgesia. However, in previous literature there are data about astrocytic expression of NR2B [44, 45]. It suggests that apart from astrocyte‐derived IL‐1β activating neuronal NR2B action, we cannot exclude the possibility that astrocytic expression of NR2B also plays a role in SIH. Future investigations should elucidate the detailed mechanisms.

In summary, our findings provided evidence that IL‐1β‐dependent, c‐JNK‐regulated astrocyte–neuron signaling pathway in PTSD‐induced hyperalgesia. We showed that PTSD‐induced dysregulation of immune responses could lead to an increase in mechanical allodynia. More GFAP activation in SPS + CFA might lead to IL‐1β production by JNK signaling. IL‐1β/IL‐1R signaling contributed to NR2B activation, amplifying pain sensation by posttranslational regulation.

**Nomenclature**
SPS: Single‐prolonged stress
CFA: Complete Freund’s adjuvant
MWT: Mechanical withdrawal threshold
GFAP: Glial fibrillary acidic proteins
IL‐1RI: IL‐1β receptor
NR2B: NMDAR2B
JNK: c‐Jun N‐terminal kinase
SIH: Stress‐induced hyperalgesia
PTSD: Posttraumatic stress disorder
NMDAR: NMDA receptor
ELISA: Enzyme‐linked immunosorbent assay.

## Author Contributions

Jian Qi wrote the main manuscript text and prepared Figures 1–6. Chen Chen prepared Figures 7–9. Qian Gao prepared Figures 10 and 11. All authors reviewed the manuscript.

## Funding

This work was supported by grants from the Shandong First Medical University Teaching Program (Grant XM2022131) and the Military Medical Service Support Capability Innovation and Generation Special Plan (Grant 20WQ021).

## Conflicts of Interest

The authors declare no conflicts of interest.

## Data Availability

The data that support the findings of this study are available from the corresponding author upon reasonable request.
